# Background modelling of diffraction data in the presence of ice rings

**DOI:** 10.1107/S2052252517010259

**Published:** 2017-08-08

**Authors:** James M. Parkhurst, Andrea Thorn, Melanie Vollmar, Graeme Winter, David G. Waterman, Luis Fuentes-Montero, Richard J. Gildea, Garib N. Murshudov, Gwyndaf Evans

**Affiliations:** a Diamond Light Source Ltd, Harwell Science and Innovation Campus, Didcot OX11 0DE, England; b Laboratory of Molecular Biology, Francis Crick Avenue, Cambridge CB2 0QH, England; c Hamburg Centre for Ultrafast Imaging, Universität Hamburg, Luruper Chaussee 149, 22761 Hamburg, Germany; d STFC Rutherford Appleton Laboratory, Didcot OX11 0FA, England; e CCP4, Research Complex at Harwell, Rutherford Appleton Laboratory, Didcot OX11 0FA, England

**Keywords:** protein structure, refinement, X-ray crystallography, ice rings, data processing, data analysis, X-ray diffraction, data quality, *AUSPEX*, *DIALS*

## Abstract

An algorithm for modelling the background for each Bragg reflection in a series of X-ray diffraction images containing Debye–Scherrer diffraction from ice in the sample is presented. The method involves the use of a global background model which is generated from the complete X-ray diffraction data set. Fitting of this model to the background pixels is then performed for each reflection independently. The algorithm uses a static background model that does not vary over the course of the scan. The greatest improvement can be expected for data where ice rings are present throughout the data set and the local background shape at the size of a spot on the detector does not exhibit large time-dependent variation. However, the algorithm has been applied to data sets whose background showed large pixel variations (variance/mean > 2) and has been shown to improve the results of processing for these data sets. It is shown that the use of a simple flat-background model as in traditional integration programs causes systematic bias in the background determination at ice-ring resolutions, resulting in an overestimation of reflection intensities at the peaks of the ice rings and an underestimation of reflection intensities either side of the ice ring. The new global background-model algorithm presented here corrects for this bias, resulting in a noticeable improvement in *R* factors following refinement.

## Abstract 2

An algorithm for modelling the background of X-ray diffraction images in the presence of ice rings is presented.

## 1. Introduction   

In macromolecular crystallography (MX), for data collected using the rotation method, a data set is typically composed of a sequence of X-ray diffraction images (Arndt & Wonacott, 1977 ▸); each image covers a fixed oscillation and, as the crystal is rotated, individual reflections enter and subsequently exit the diffracting condition. Integration programs, such as *MOSFLM* (Leslie, 1999 ▸), *XDS* (Kabsch, 2010 ▸), d*TREK (Pflugrath, 1999 ▸), *HKL*-2000/*DENZO* (Otwinowski & Minor, 1997 ▸) and *DIALS* (Waterman *et al.*, 2013 ▸), are used to predict where each Bragg reflection will appear on the detector and then to provide an estimate of the intensity of each reflection. The simplest method for computing the reflection intensities is *via* summation integration; most integration programs provide an implementation and, whilst the details may differ, the procedure is generally the same.

(i) Firstly, the location and extent of each reflection on the detector is predicted and, for each reflection, pixels are assigned as either *foreground* or *background* depending on whether or not they are predicted to contain signal from the Bragg reflection.
(ii) The background under the reflection peak is then estimated. Since it is not possible to measure the background under the peak directly, the background in the foreground pixels is estimated from the surrounding background pixels. As such, a model of the background is required and the model is fitted to the background pixel data.
(iii) Finally, the reflection intensity is estimated by summing the total counts in the foreground region and subtracting the sum of the estimated background counts.

Traditionally, in most integration programs simple background models have been employed; a major reason for this is the necessity of having a computationally efficient implementation since the background needs to be estimated for a large number of reflections in each data set. Furthermore, the best way to model the general reflection background is not always obvious since the background varies considerably between data sets. As such, the background under each reflection peak is often assumed to be a constant value (Kabsch, 2010 ▸) or a plane with a small gradient (Rossmann, 1979 ▸; Otwinowski & Minor, 1997 ▸; Leslie, 1999 ▸). In *DIALS*, either a constant or planar background can be used (Parkhurst *et al.*, 2016 ▸). In a typical MX X-ray diffraction data set, individual reflections extend over a small number of pixels; therefore, this assumption often holds true – the local background is fairly flat – and these simple models have been employed with great success for many years (Diamond, 1969 ▸; Otwinowski & Minor, 1997 ▸; Leslie, 1999 ▸; Kabsch, 2010 ▸).

Whilst such a simple background model may be appropriate in the majority of cases, particularly for well measured data, it is not applicable where the background changes significantly over the extent of a single reflection peak. In such cases a flat or planar background model is likely to provide an inaccurate estimate of the background in the reflection-peak region. Large variation in the background counts can be the result of various effects such as scattering from the cryostream nozzle or, in serial crystallography, from the linear jet that transports crystals into the beam, which creates a streak of diffraction perpendicular to the jet direction. Large variation is also often seen around the backstop; however, these reflections are usually omitted from processing owing to their large Lorentz factor. Perhaps the most common pathology seen in diffraction images resulting in a large variation in background counts is the presence of water ice rings (Mitchell & Garman, 1994 ▸). A detailed description of the theoretical manifestation of cubic and hexagonal ice, the most common forms, in diffraction images can be found in Thorn *et al.* (2017 ▸). In practice, when cubic ice diffraction is observed, hexagonal ice diffraction is also observed (Fuentes-Landete *et al.*, 2015 ▸).

**Figure 1:**
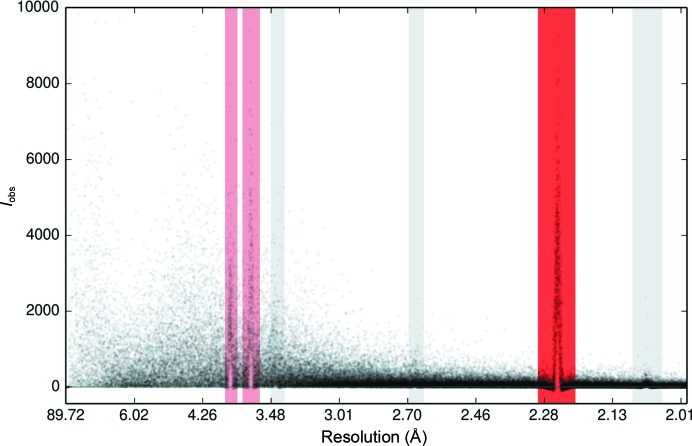
Intensity *versus* resolution for a data set with strong ice rings. Such plots can be readily generated by *AUSPEX* (Thorn *et al.*, 2017 ▸). The points show the intensities for individual reflections. The characteristic ice-ring resolutions are shown here in grey, with automatically detected ice rings flagged in red. Spikes in reflection intensity are observed at ice-ring resolutions, indicating bias in the background determination.

If the background is assumed to be locally flat, but ice rings are present, the reflection intensities will be systematically biased. The effect on the background estimation caused by the presence of ice rings can be readily seen by plotting the scaled reflection intensities as a function of resolution, as shown in Fig. 1 ▸. This plot shows a large spike in the reflection intensities at ice-ring resolutions, with a drop in the reflection intensities either side of the ice ring; *i.e.* the presence of ice rings causes both systematic overestimation and underestimation of the reflection intensities at characteristic resolutions. Indeed, at high resolution, where the true reflection intensities are very small, the positive systematic bias in the background estimate causes the average reflection intensity to be less than zero at resolutions immediately either side of the ice rings.

**Figure 2:**
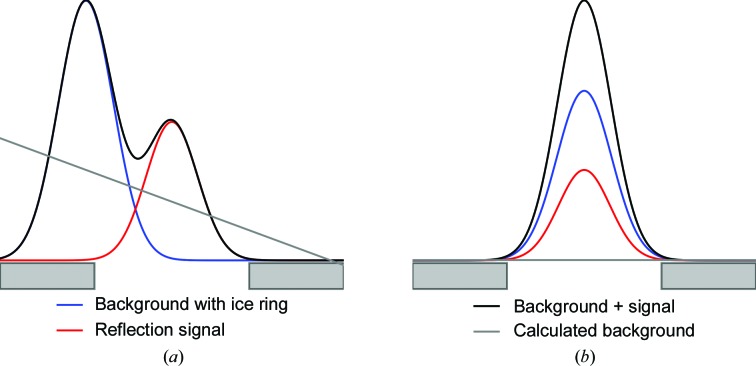
Illustration of the effect of ice rings on the background determination when a simple plane model is employed. The shaded rectangles indicate the background pixels used to estimate the background. When the reflection is centred on the tail of the ice ring (*a*) the background is overestimated. When the reflection is centred on the peak of the ice ring (*b*) the background is underestimated.

The cause of this effect can be understood by considering the application of a simple background model to a reflection positioned close to an ice ring, as illustrated in Fig. 2 ▸. As the ice ring intrudes into the background region of the reflection shoebox, the background level in the reflection-peak region is overestimated owing to the higher valued counts from the ice ring. When the reflection foreground covers the peak of the ice ring, then the background region of the reflection shoebox contains pixels with fewer counts than should be modelled in the reflection peak. Consequently, the background in the reflection peak will be underestimated and the reflection intensity will be overestimated. This leads to many reflections being rejected as outliers during data reduction, resulting in a loss of information. This effect is more pronounced for sharper ice rings; however, on average, fewer reflections will be affected than in the case of more diffuse ice rings which cover a larger resolution range.

In most integration programs, the handling of ice rings and other complex background features is problematic, and proper modelling is rarely attempted. However, some programs, such as *MOSFLM* and *XDS*, do provide parameters to exclude reflections within a user-specified resolution range. Therefore, in these programs reflections falling on ice rings can be easily excluded from the processing if desired, which usually results in a loss of otherwise potentially useful information. In d*TREK and *HKL*-2000/*DENZO* (Otwinowski & Minor, 1997 ▸) parameters are provided to remove reflections whose background counts vary excessively; however, again, this will result in information loss. It is often the case that the reflections recorded on ice rings are handled during scaling rather than integration. This is particularly the case at higher resolution where ice rings may not be immediately visible on single detector images. Scaling programs such as *AIMLESS* (Evans & Murshudov, 2013 ▸) have outlier-handling routines that exclude intensity measurements that are not consistent between symmetry-equivalent reflections; additionally, a resolution range can be set to exclude reflections from the scaling. Programs such as *CTRUNCATE* (Winn *et al.*, 2011 ▸), *phenix.xtriage* (Zwart *et al.*, 2005 ▸) and *AUSPEX* (Thorn *et al.*, 2017 ▸) can be used to automatically determine, from the scaled reflection data, whether the data have been contaminated by ice rings.

An attempt to handle ice rings external to the integration program is described by Chapman & Somasundaram (2010 ▸). They describe a method to subtract the ice-ring intensity from the raw image data as a pre-processing step before integration. However, this approach is not ideal since the statistics of the data will be altered. Furthermore, the shape of the ice rings is assumed to be radially Gaussian with resolution and perfectly circular, which may not be the case in practice. The data as recorded by a photon-counting detector are ‘count data’, which are well modelled by a Poisson distribution. The Poisson distribution is discrete and only valid for positive pixel counts. Subtracting the background prior to the integration will result in the data no longer being Poisson-distributed; some pixels may contain negative counts and others may contain a non-integer number of counts. This will render assumptions about the statistical properties of the data in the integration program invalid and will have an impact on the estimation of the errors in the intensities. For this reason, the ice-ring background should be modelled explicitly during the reflection-integration step.

## 2. Algorithm   

We describe a new algorithm for modelling the X-ray diffraction background in the presence of ice rings. The algorithm consists of two distinct steps: firstly a global model of the background at each image pixel is generated, and the model is then fitted locally and independently for each predicted reflection in the data set. The implementation of the algorithm and program usage in *DIALS* is given in Appendix *A* [App appa].

### 2.1. Global background model   

In the current implementation in *DIALS* (Waterman *et al.*, 2013 ▸), we restrict ourselves to considering a static model that is applied to reflections over the entire rotation scan. For the static background-model algorithm, we assume that the shape of the background model remains fairly stable across all of the images in the data set. We assert that in the case where the background is contaminated with ice rings, an approximate model should perform better than a flat background model; this therefore represents an improvement in the handling of data with a complex background.

The global background model is calculated as the mean value at each pixel averaged over all images in the data set. This method for generating the global background model is computationally efficient and simple to compute; care needs to be taken to ensure that the inclusion of outlier pixels does not cause the background model to be distorted. In this context, outlier pixels are considered to be pixels which contain intensity from predicted reflections as well as unmodelled intensity, which may come from reflections whose extent is badly predicted, zingers (random spikes in intensity from, for example, cosmic rays) or other sources.

Intensity from predicted reflections is handled by generating a mask for each image delineating the foreground and background for each reflection, and then using only background pixels for the global background model. The mask contains True where the pixels are predicted to only contain background counts and False where they are predicted to contain intensity from predicted reflections. Once the process concludes and a mean value is computed at each pixel, the number of images contributing to the mean for each pixel is calculated. A second pixel mask is then generated containing True where the number of contributed images is greater than some user-specified value (by default ten) and False otherwise. In this way, pixels where only a small number of images have contributed are excluded. Where the number of images in the data set is less than ten, the minimum number of required images is reduced; however, the method is most effective where a larger number of images is available.

In order to ensure that the model is not affected by outliers caused by unmodelled intensity, a number of filters are applied to the mean image to produce the final background model as follows.

(i) Firstly, the mean image and mask are transformed into a polar image such that columns in the transformed image correspond to lines of constant resolution. This requires that each pixel in the raw untransformed image is mapped onto the transformed grid. In the implementation described here, this is performed by computing the overlap of each pixel in the raw image with the transformed grid and then using a polygon-clipping algorithm (Sutherland & Hodgman, 1974 ▸) to compute the overlapping area between the pixel and the grid. The fractional overlap is then used to determine the fraction of counts in each pixel that is distributed to each grid point in the transformed image. The number of counts in the raw and transformed images is then conserved. The benefit of applying filtering to this transformed image rather than the raw mean image is that, in the case of ice rings in particular, the background is likely to vary less along lines of constant resolution. Therefore, the variation along columns in the transformed image is likely to be small and most variation will occur along the rows, which correspond to increasing resolution. The polar transform will tend to sample pixels at high resolution less finely than at low resolution, resulting in smoothing along lines of constant resolution.
(ii) Median filtering is then applied to the transformed image such that for each pixel the median of the neighbouring *N* (by default ten) pixels along each column (wrapped at the column ends) is used; this has the effect of removing the effect of unwanted outliers, in particular high-valued pixel outliers.
(iii) The transformed image will contain pixels that are masked out; these need to be filled in order to provide full coverage of the detector for the background model. Again, since the image has been transformed and the variation along columns is small, the missing pixel values can be filled using a simple iterative diffusion algorithm based on the application of Laplace’s equation with Dirichlet boundary conditions, whereby missing pixels are iteratively filled with the values derived from adjacent pixels until convergence is achieved.
(iv) Finally, the polar image is transformed back *via* the same polygon-clipping process, with the counts in the transformed image being redistributed to the image in the original coordinate system. Application of this gridding procedure will result in some additional smoothing in the processed image. The final result is a smoothly varying global background model.

### 2.2. Maximum-likelihood fitting for each reflection   

The background is fitted to each reflection locally and independently by simply scaling the background model to fit the counts in the background region of the reflection in question. The pixel counts are assumed to be drawn from a Poisson distribution. For each pixel *i* in the reflection background, consisting of *N* pixels, the probability of observing *c_i_* counts, given the background model *b_i_* scaled by the parameter *B*, is
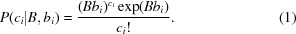
The value of the parameter *B* is then estimated *via* maximum likelihood by considering the joint probability distribution over the *N* pixels, 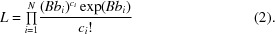
Using the log likelihood and taking derivatives with respect to the scale parameter, ∂log(*L*)/∂*B* = 0, results in a very simple and computationally efficient equation for the scale factor *B* for each reflection, which is simply
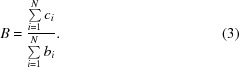
However, this equation for the scale factor is not resistant to pixel outliers in the reflection background, which must be handled to ensure that the background estimates are reliable. Since the data are Poisson-distributed, a principled approach to the modelling of the background in the presence of pixel outliers would be to use a robust generalized linear model (GLM) algorithm (Parkhurst *et al.*, 2016 ▸); however, whereas the robust GLM algorithm can be made computationally efficient for the case of a flat background model, the algorithm proved to be difficult to optimize in the case of more complex models. Computational efficiency is a requirement of any background-modelling algorithm in integration since the background needs to be estimated for a large number of reflections in each data set. Therefore, a simpler approach was taken in this case. The approach used here was to use the Anscombe variance-stabilizing transform for a Poisson distribution (Anscombe, 1948 ▸) given by *y* = 2(*x* + 3/8)^1/2^. This transforms the Poisson-distributed data such that they are approximately normally distributed with a variance of 1. This transformation is biased where the Poisson scale parameter is very small (<4); however, in the case of data where the background is contaminated with ice rings the background is generally much larger and this approximation may be used. The robust estimation is then performed using the Huber weighting function (Huber, 1964 ▸) such that for a pixel *i*, with transformed value *y_i_*, predicted value μ*_i_*, variance *v_i_* and residual *r_i_* = (*y_i_* − μ*_i_*)/*v_i_* ^1/2^, the pixel weighting *w_i_* will be given by
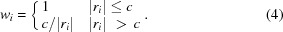



This weighting function has the effect of damping values outside a range defined by the tuning constant *c*, whose default value is 3 (*i.e.* transformed pixel values greater than three standard deviations from the mean are damped). The quasi-likelihood equation implementing this robust algorithm is then solved using iteratively reweighted least squares, as described in Appendix *B* [App appb].

## 3. Analysis   

### 3.1. Experimental data   

In order to evaluate the effect on the quality of processed data when there are prominent ice rings in the X-ray background, some data sets were selected from the Joint Centre for Structural Genomics (JCSG; Gabanyi *et al.*, 2011 ▸). Whilst the method is also applicable to data collected using other detectors, only data sets collected using a Dectris PILATUS detector (Henrich *et al.*, 2009 ▸) were considered for analysis. Data sets were chosen manually by inspecting a plot of the intensity *versus* resolution using *AUSPEX* (Thorn *et al.*, 2017 ▸); those data sets showing a noticeable systematic bias at ice-ring resolutions were used (see Fig. 6). Two data sets (PDB entries 4mjg and 4puc) were identified in which reflections in entire resolution ranges corresponding to ice rings had been discarded in deposition; in the following analysis, the data were processed without omission. 13 data sets that showed ice-ring pathologies and which were successfully processed using *DIALS* inside the *xia*2 (Winter, 2010 ▸) automatic processing pipeline were used in the analysis. Table 1 ▸ shows the data sets used in more detail, giving the known space group and resolution. Details of the data processing, data reduction and refinement are given in Appendix *C* [App appc].

### 3.2. Refinement results   

**Figure 3:**
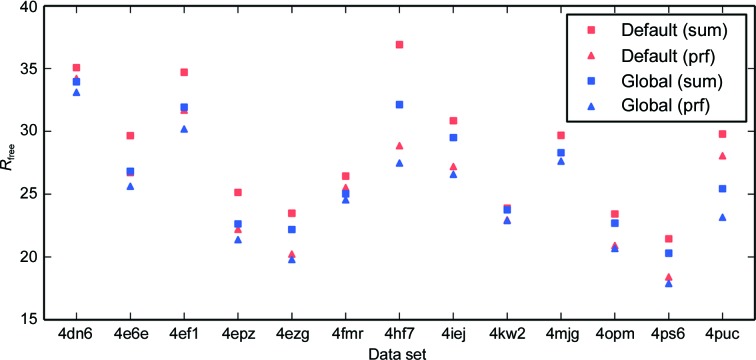
The *R*_free_ after refinement using *REFMAC*5 for both summation integration and profile-fitting integration using both the default background algorithm assuming a flat background model and the global background-model algorithm. In all cases, the global background model results in an improvement in the *R*_free_ factors from refinement. Using profile fitting (prf) instead of summation (sum) also results in an improvement in *R*_free_ in each case.

The *R*_work_ and *R*_free_ statistics reported by *REFMAC*5 (Murshudov, 2011 ▸) for each data set as processed with both the default background algorithm and the global background algorithm are shown in Table 1 ▸; additionally, *R*_free_ for each data set is shown in Fig. 3 ▸. The improvement in *R*_work_ and *R*_free_ is shown for both summation-integrated data and profile-fitted data. It can be seen that in each case both *R*_work_ and *R*_free_ are reduced by the use of the global background-model algorithm over the default background algorithm. An improvement is seen when the data are processed using both summation integration and profile fitting. In some cases (for example PDB entries 4kw2 and 4opm) the improvement is minor; however, in others, such as PDB entry 4puc, the improvement in the refinement *R* factors is dramatic, with *R*_free_ being reduced by 4.9%. In the case of PDB entry 4puc, as previously reported, reflections from entire resolution ranges around ice rings were omitted in the deposited data (the completeness of the deposited data was 78.1%; the completeness of the data processed here is 99.3%). In general, most data sets see a moderate improvement in the *R*_free_; the mean improvement in *R*_free_ across all data sets was 2.0% when using summation integration and 1.1% when using profile fitting. Profile fitting also consistently results in lower *R*_work_ and *R*_free_ values than summation integration, and the improvement in *R*_work_ and *R*_free_ when using the new global background model algorithm is slightly lower than the improvement observed with data processed using summation integration.

### 3.3. Case studies   

**Figure 4:**
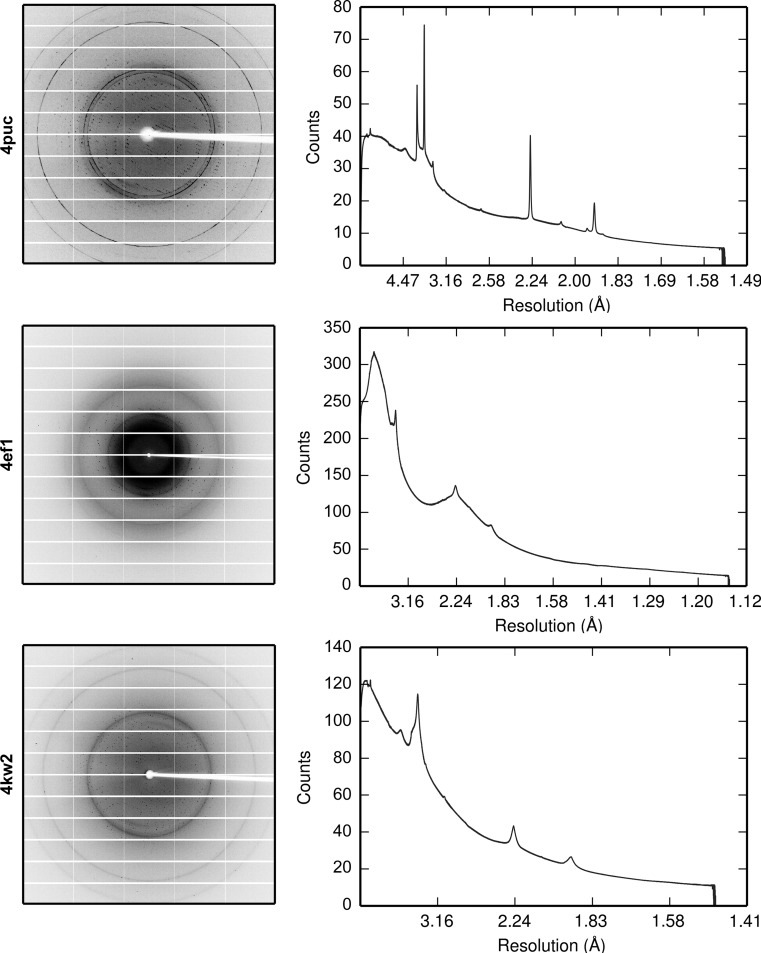
The first image for each data set and the corresponding average background *versus* resolution for 4puc (top), 4ef1 (middle) and 4kw2 (bottom). Ice rings are visible as discrete or diffuse rings. Note the irregular ice rings in the diffraction image of 4puc resulting from the dominating ice-crystal orientation. Also note that the maximum resolution on each image differs; therefore, the ice rings are not always in the same place on the detector.

Of the 13 JCSG data sets processed above, three were selected for more detailed analysis. The first image and the average background *versus* resolution for each of the data sets is shown in Fig. 4 ▸. The data sets were chosen as follows.

(i) PDB entry 4puc. This data set shows an example of very strong and prominent ice rings. The ice rings in this data set are narrow and the three inner rings corresponding to hexagonal ice rings can be clearly distinguished in the diffraction images. Handling reflections falling on these ice rings is likely to be a challenge for current background-modelling algorithms. Each image in the data set covers a rotation of 0.25°. This is a MAD data set consisting of three sweeps at different wavelengths. In the data processing, all three sweeps were used and merged together.
(ii) PDB entry 4ef1. This data set shows a moderate improvement in the *R* factors. The data set has ice rings from nanocrystalline cubic ice. Each image in the data set covered a rotation of 0.3°.
(iii) PDB entry 4kw2. This data set shows the smallest improvement in the *R* factors. The data set has ice rings from nanocrystalline cubic ice. Each image in the data set covered a rotation of 0.5°. This is a MAD data set consisting of 18 sweeps at different wavelengths. In the data processing, all 18 sweeps were used and merged together.

### 3.4. Pixel statistics   

**Figure 5:**
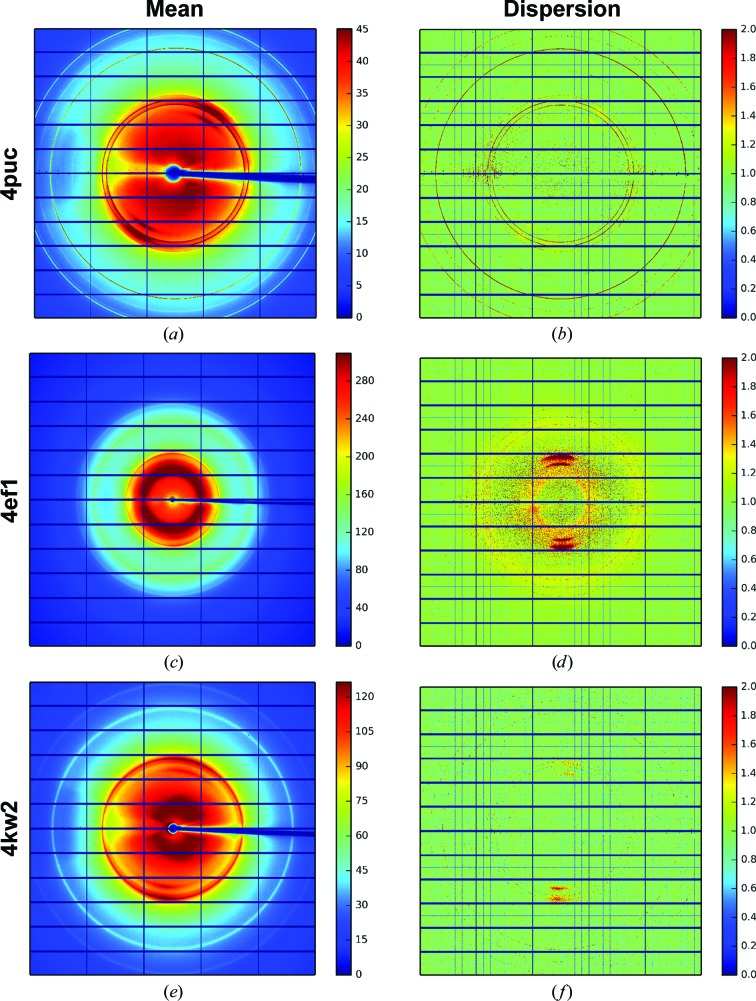
The mean and dispersion images for 4puc (*a*, *b*), 4ef1 (*c*, *d*) and 4kw2 (*e*, *f*). The mean image is the mean value at each pixel through the image stack; the index of dispersion image shows the variation across the data set at each pixel. In the mean image the ice rings are clearly visible. The dispersion images also show the structure of the detector. The boundaries between the detector chips are visible as lines of pixels that are under-dispersed relative to a Poisson distribution. This is owing to the use of virtual pixels between chips that share counts and whose values are therefore correlated.

During the creation of the global background model, the mean, variance and index of dispersion (variance/mean) are calculated independently for each pixel across all images in the rotation scan. Note that pixels predicted to contain intensity from reflections are not used in the calculation of these images. The mean and index of dispersion are shown for each data set in Fig. 5 ▸. From a qualitative inspection, the mean background image visually resembles a smoothed version of the raw image data shown in Fig. 4 ▸. The dispersion images, however, indicate that the variation in the background is not uniform across the detector surface. In particular, background pixels not containing ice rings appear to vary very little across the rotation scan, as indicated by the index of dispersion being close to 1.0. By contrast, pixels containing ice rings appear to show much greater variation across the scan, with an index of dispersion of greater than 2.0 in some cases. This appears to indicate that the intensity of the ice-ring background varies much more than the general background counts.

### 3.5. Intensity *versus* resolution   

**Figure 6:**
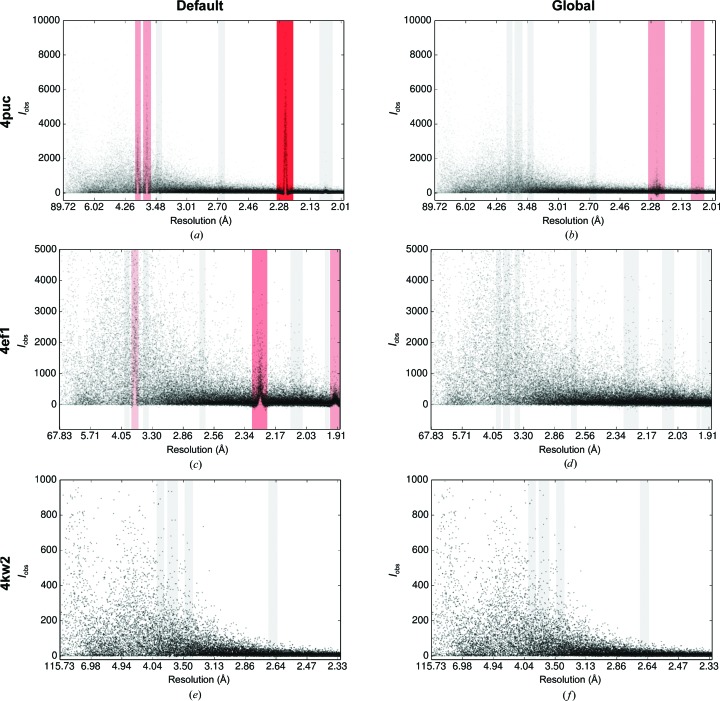
The intensity *versus* resolution of reflections processed using the default background algorithm (left) and the new global background-model algorithm (right) for data sets 4puc (*a*, *b*), 4ef1 (*c*, *d*) and 4kw2 (*e*, *f*). All plots were generated by *AUSPEX* (Thorn *et al.*, 2017 ▸). The points represent the individual intensities and the vertical bars show the resolutions at which ice rings may be found. The red bars refer to suspected ice rings found by *AUSPEX*.

Fig. 6 ▸ shows the intensity plotted against resolution for all reflections in each data set with the default background algorithm and the new global background-model algorithm. In each case, for the default background algorithm it can be seen that the reflection intensities at ice-ring resolutions suffer from systematic bias. This is shown as spikes in the intensity at ice-ring resolutions. These spikes are owing to the background of reflections lying at ice-ring resolutions being underestimated. Thus, the reflection intensity is overestimated. Small dips in intensity can be seen either side of the ice rings, showing how the background is overestimated as the ice ring intrudes into the background region of the reflection shoeboxes, thereby causing the reflection intensities to be underestimated. This is particularly noticeable for data set 4puc, where the shift is dramatic. Data set 4ef1 shows a moderate increase in reflection intensities at the ice-ring resolutions; data set 4kw2 only shows a fairly minor shift visible in the ice ring at 3.7 Å.

For the new global background-model algorithm, the intensity estimates appear to be greatly improved. For the 4ef1 and 4kw2 data sets, the spikes at ice-ring resolutions are completely absent, indicating that the systematic bias in the intensity estimates has been reduced relative to the bias for the default background algorithm. For the 4puc data set, which was the most challenging data set, there is some improvement; however, peaks are still present at some ice-ring resolutions. This is owing to the ice-ring background being sharp and irregular with time-dependent variation throughout the data set. Taken together, these conditions provide a difficult modelling challenge. The algorithm computes the global background model over a number of images; therefore, the algorithm will tend to perform worse where there are large time-dependent variations in the background shape. Nevertheless, 4puc showed the best improvement in refinement *R* factors, as shown in Table 1 ▸.

### 3.6. Moments of *E* and *R*_free_ *versus* resolution   

**Figure 7:**
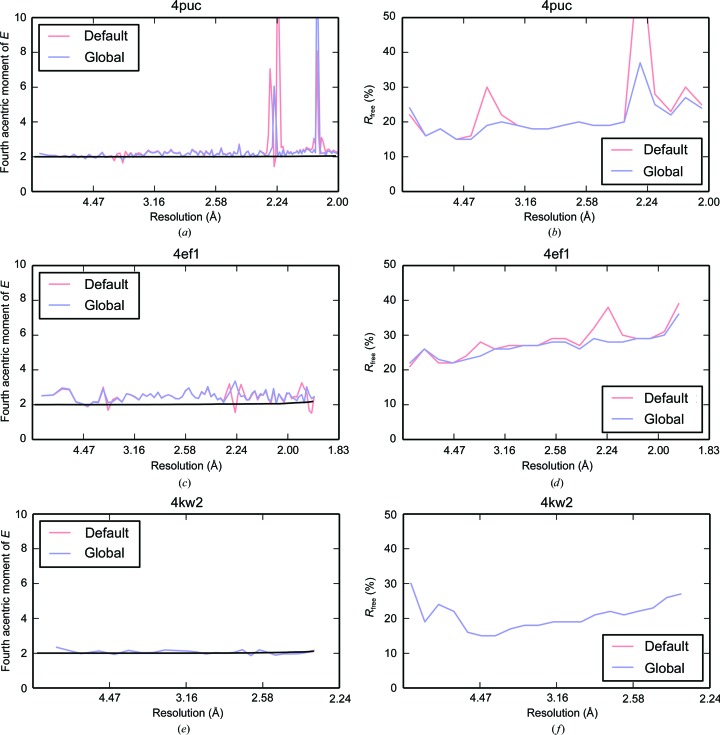
The fourth acentric moments of *E*, the normalized structure factors, *versus* resolution for data sets (*a*) 4puc, (*c*) 4ef1 and (*e*) 4kw2. The red line indicates the default background algorithm and the blue line indicates the new global background-model algorithm. The expected value for untwinned data is shown by the theoretical curve in black. The *R*_free_ *versus* resolution for data sets (*b*) 4puc, (*d*) 4ef1 and (*f*) 4kw2. The red line indicates the default background algorithm and the blue line indicates the new global background-model algorithm.

The left panel in Fig. 7 ▸ shows the fourth acentric moments of *E*, the normalized structure factors, for each data set processed with both the default background algorithm and the new global background-model algorithm as produced by *CTRUNCATE* (Winn *et al.*, 2011 ▸). For error-free data, the fourth moment takes on a value of 2 for untwinned data and 1.5 for perfectly twinned data (Stein, 2007 ▸). When the variances on the intensities are taken into account, the value of the moments is inflated by σ(*I*)^2^/〈*I*〉^2^, as described in Appendix *D* [App appd]; this is shown by the theoretical curve in Fig. 7 ▸, which was generated by the *Phaser* program (McCoy *et al.*, 2007 ▸). The right panel in Fig. 7 ▸ shows *R*_free_ *versus* resolution as reported by *REFMAC*5 (Murshudov *et al.*, 2011 ▸).

The moment plots seem to mirror those seen in the intensity *versus* resolution plots. Data set 4puc shows large deviations from the expected value of 2 at ice-ring resolutions with the default background algorithm. After application of the new global background-model algorithm the moments, whilst better behaved, still show the effect of the ice rings. For 4ef1, the moments differ at ice-ring resolutions for data processed with the default background algorithm and data processed with the new global background algorithm. However, the variation is small relative to the noise. For 4kw2, which showed little improvement after application of the global background-model algorithm, the ice rings seem to have very little effect on the moments. For data processed with both the default background algorithm and the new global background-model algorithm, the moments follow the expected theoretical curve. It is clear that the moments are not always a clear indicator of ice rings in the data. In particular, a mild pathology may not alter the moments such that the effect is visible through the noise; however, the effect may be visible for more prominant ice-ring cases, such as for data set 4puc.

A plot of *R*_free_ against resolution provides a better indication of the effect of ice rings on the data; however, it is only available after refinement. For data sets 4puc and 4ef1 the effect of applying the new global background-model algorithm is immediately clear: the *R*_free_ at ice-ring resolutions is drastically decreased relative to the *R*_free_ using the default background algorithm. As also shown in the previous analysis, the difference observed in the *R*_free_ for data set 4kw2 is negligable. Inspecting this plot may give some indication as to the effect of ice rings on the data, particularly for data containing very prominant ice rings, such as data set 4puc.

### 3.7. Application to data with no ice rings   

As a control, a weak thaumatin data set collected on beamline I04 at Diamond Light Source and known to contain no visible ice-ring pathologies (Winter & Hall, 2014 ▸) was processed to ensure that the new global background-model algorithm gives good results in the case of well collected data. The average background over all resolution ranges is less than one count per pixel; there is also a low incidence of outliers in the background pixels. The data set was processed to a resolution of 1.2 Å using the same procedure as described in Appendix *C* [App appc].

It was found that the use of the global background-model algorithm for this data set resulted in no difference in the refinement *R* factors. Refinement with *REFMAC*5 resulted in the same *R*_free_ for data processed with both the default background algorithm and the new global background-model algorithm. When summation integration was used the *R*_free_ was 18.1% in each case and when profile fitting was used the *R*_free_ was 17.4% in each case, thereby further illustrating the trend seen previously, where a reduction is *R*_free_ is observed when using profile fitting over summation integration. Furthermore, plots of the moments with resolution and the *R*_free_ with resolution showed no difference between data processed using the default background algorithm and the global background-model algorithm.

## 4. Conclusion   

The use of a new global background-model algorithm for the processing of X-ray diffraction data in the presence of ice rings is presented. Traditional approaches to background modelling as implemented in current integration programs do not adequately cope with the task of modelling reflection background that is not well described by either a constant or a plane with a small slope. Consequently, these methods introduce systematic bias into the background estimation for reflections whose integration shoeboxes overlap with ice rings. This bias renders the majority of reflection intensities at certain resolutions unreliable if the data set is contaminated by ice diffraction. At the peak of an ice ring, reflection intensities tend to be overestimated owing to an underestimation of the reflection background. To either side of the ice ring, reflection intensities tend to be underestimated owing to an overestimation of the reflection background. The use of a simple global background-model algorithm has been shown to correct for these issues. Modelling the background in the presence of ice rings is challenging; however, correct modelling can have a noticeable effect on the downstream data processing. Finally, it is important to note that whilst it is possible to correct for the effect of ice rings in data-processing software, better results can be obtained by ensuring that samples are not contaminated with ice to begin with.

### 4.1. Future improvements   

The current implementation uses a simple static background model which is applied to each image in the data set. A future enhancement to the algorithm may be to employ a scan-varying global background model, where the model is allowed to vary over the course of the rotation scan. Additionally, the algorithm may be enhanced by generating a number of models (for example a flat and planar model as well as a curved model based on the global background model) and fitting to each reflection, with the model then being selected by a model-selection algorithm; for example, the Akaike Information Criteria (AIC; Akaike, 1973 ▸). 

## Figures and Tables

**Table 1:** A list of JCSG data sets with ice-ring pathologies The improvement in *R*_free_ reported by *REFMAC*5 for data integrated using the global background-model algorithm over data integrated with the default background algorithm is given for profile-fitted intensities (prf) and summation intensities (sum). For each data set, the same set of integrated reflections were used for the different sets of processing. Here, Δ = *R*_default_ − *R*_global_; a positive value indicates an improvement using the global background-model algorithm. For bevity, columns with data from the default background algorithm are labelled ‘D’ and columns with data from the global background algorithm are labelled ‘G’. The completeness is shown for the data processed (*DIALS*) and the completeness reported in the PDB. For 4puc, an *R*_free_ of 19.98% is reported in the PDB. For this data set, reflections falling on ice rings were excluded from processing, resulting in low completeness. Refining the subset of reflections present in the deposited data processed using the new background algorithm against the deposited structure resulted in an *R*_free_ of 19.46%.

				Completeness (%)		*R*_free_ (sum) (%)			*R*_free_ (prf) (%)		
PDB code	Space group	Resolution (Å)	Multiplicity	*DIALS*	PDB	D	G	Δ	D	G	Δ
4dn6	*P*4_2_2_1_2	2.80	12.6	100.0	99.0	35.1	34.0	1.1	34.2	33.1	1.1
4e6e	*P*3_2_21	2.12	12.5	99.9	99.6	29.7	26.8	2.8	26.7	25.6	1.1
4ef1	*P*12_1_1	1.90	3.4	98.0	97.8	34.7	31.9	2.8	31.7	30.2	1.5
4epz	*C*222_1_	1.68	4.2	98.5	98.0	25.1	22.6	2.5	22.2	21.4	0.8
4ezg	*P*2_1_2_1_2_1_	1.50	5.3	98.8	99.0	23.5	22.2	1.3	20.2	19.8	0.4
4fmr	*P*12_1_1	2.25	6.9	97.2	97.9	26.4	25.0	1.4	25.5	24.6	1.0
4hf7	*C*222_1_	1.77	9.8	99.7	98.2	36.9	32.1	4.8	28.9	27.5	1.4
4iej	*P*6_1_22	1.45	8.3	100.0	99.8	30.9	29.5	1.3	27.2	26.6	0.6
4kw2	*F*432	2.32	110.4	100.0	99.8	23.9	23.8	0.1	23.0	22.9	0.1
4mjg	*P*3_2_21	2.65	8.2	99.9	88.7	29.7	28.3	1.4	28.3	27.6	0.7
4opm	*C*121	1.70	7.0	99.6	97.1	23.4	22.7	0.7	20.9	20.7	0.2
4ps6	*P*12_1_1	1.25	6.2	93.7	85.8	21.5	20.3	1.2	18.4	17.9	0.5
4puc	*P*2_1_2_1_2_1_	2.00	9.2	99.3	78.1	29.8	25.4	4.4	28.1	23.2	4.9

**Table 2:** Definitions of the mathematical quantities used

Item	Definition
**y**	The vector of pixel values transformed using the Anscombe transform. The value of the *i*th pixel is given by *y_i_*.
**X**	The design matrix describing the linear model. A row in the design matrix is given as **x** *_i_*; each row gives the explanatory variables for pixel *i*.
**β**	The vector of model parameters which are estimated from the quasi-likelihood algorithm. The *j*th parameter is given by β_*j*_.
**μ**_*i*_	The estimated pixel values. These depend on the value of the parameters as **μ** = **Xβ**. The pixel value estimate for the *i*th pixel is given by μ_*i*_.
*r_i_*	The residual for the *i*th pixel given by *r_i_* = (*y_i_* − μ*_i_*)/*v_i_* ^1/2^.
**W**	The diagonal matrix of weights, where the *i*th diagonal element is given by *w*(*r_i_*), the weight for the *i*th residual.
ρ(*r_i_*)	The robust function of residuals.

## Appendix A Algorithm usage in *DIALS*

The algorithm was implemented in C++ for use within *DIALS*. The global background-model calculation is implemented as a separate program, *dials.model_background*. This program generates a file, background.pickle, which contains the computed global background model. It also generates a series of diagnostic images which can be used to inspect the properties of the data set and the quality of the background model prior to integration. These include the minimum and maximum value at each pixel in the data set and the mean, variance and index of dispersion (variance/mean) images. The mean image is used to generate the background model and the index of dispersion image is useful for evaluating the variation in the background at each pixel. Recalling that for a Poisson distribution the index of dispersion *D* = variance/mean = 1, then values significantly greater than 1.0 will indicate large variation in the counts for that pixel over the course of the data set. An image of the final background model is also generated; viewing this allows a qualitative assessment of whether the generated model is appropriate for the data. The mean image or generated background model could also be used to provide automatic ice-ring detection.

The background model is then applied in the *dials.integrate* program by setting the background.algorithm=gmodel user parameter to perform integration using the global background-model algorithm. The robust or nonrobust fitting algorithm can be selected *via* a user parameter depending on what is most appropriate for the particular data set. Currently, the input experiments file must contain the profile information generated from a successful integration run; therefore, an initial integration run is required before performing the global background modelling. In future versions of *DIALS* these steps may be applied together. Sample program usage is shown below.

## Appendix B Robust M-estimator for background scale factor

The terms used in the following equations are defined in Table 2 ▸.

In robust estimation, M-estimators minimize a function of residuals of the form

The value of each residual *r_i_* depends at each iteration on the value of the parameter estimates, **β**. Taking derivatives with respect to each parameter, β_*j*_, gives

Weights are then defined as

The equation to be solved then becomes

The parameter estimates can be found at each iteration as

In order to fit the global background model, a single scale factor is used. The design matrix **X** then has a single column. Therefore, each iteration can then be simplified to

In our implementation, the Huber function is used, which gives the robust function of residuals as

This results in the following weighting function:

## Appendix C Data processing, reduction and refinement

Aside from the choice of background-model algorithm, the details of the processing were identical in each case. Each data set was processed with *xia*2 (Winter, 2010 ▸) using *DIALS* (Waterman *et al.*, 2013 ▸) as the data-analysis engine. The integrated experiments.json file produced by *xia*2 after integration was then passed to a new program (*dials.model_background*), which was used to compute the global background model. The data were then integrated again, first using the default background algorithm and then using the global background-model algorithm. In each case the data were integrated using summation integration and profile fitting. Reflections falling on ice rings were not excluded from the data processing. For data sets composed of more than one sweep, each sweep was integrated separately.

The data were processed using the *DIALS* pipeline as follows, where ${PATH_TO_IMAGES} is a placeholder for the path to the directory containing the image data.

The procedure for re-integrating the data using the global background-model algorithm and again with the default background algorithm is shown as follows, where ${ORIGINAL_EXPERIMENTS} is a placeholder for the path to the integrated experiments.json file from the *xia*2 processing.

The data reduction was performed using *POINTLESS* (Evans, 2006 ▸), *AIMLESS* (Evans & Murshudov, 2013 ▸) and *CTRUNCATE* (Winn *et al.*, 2011 ▸), specifying the known space group and the resolution as reported in the PDB entry for each data set. For data sets composed of more than one sweep, the data sets were scaled and merged together in *AIMLESS* to produce a single merged MTZ file. A free set of reflections for cross-validation in refinement was then selected using the *FREERFLAG* program. The *UNIQUEIFY* script in the *CCP*4*i* GUI application (Winn *et al.*, 2011 ▸) was used to ensure that the same free set was used for the processing of all instances of the same PDB entry. Prior to refinement, the coordinates in the PDB file for the data set were randomized using *PDBSET* (Winn *et al.*, 2011 ▸) with a maximum noise level of 0.4 Å to ensure that there was no bias in the refinement and *R*_free_ calculation. Finally, each data set was refined to convergence against the randomized structure using *REFMAC*5 (Murshudov *et al.*, 2011 ▸).

A complete script detailing the data-reduction and refinement steps is shown below, where ${PDBID}, ${SPACEGROUP} and ${RESOLUTION} are placeholders for the known PDB identifier, space group and resolution, respectively.

## Appendix D The effect of noise on the intensity moments

The observed reflection intensity *I*_o_ is the sum of the true intensity *I*_t_ and a noise contribution *n*, such that *I*_o_ = *I*_t_ + *n*. Therefore, the first and second moment of *I*_o_ can be written as

The normalized moment can then be written as the following ratio:

If we denote *k* = var(*I*_t_)/〈*I*_t_〉^2^, then the above ratio can be written as

The value of *k* depends on the distribution of true intensities. For single-crystal intensities without statistical peculiarities such as twinning and pseudo-translation, *k* = 1. For mero­hedral twinning, *k* = 1/2. For *n*-fold twinning, *k* = 1/*n*. Therefore, for single-crystal, untwinned data the ratio is given by

Assuming in each case that there is no correlation between the signal and the noise [*i.e.* cov(*I*_t_, *n*) = 0], the following hold.

(i) If the mean noise 〈*n*〉 is 0 and the variance of the noise var(*n*) is close to zero, then
  Typically, at low resolution, where reflection intensities are large relative to the noise, the moments tend to this value.
(ii) If the mean noise 〈*n*〉 is nonzero and the variance of the noise var(*n*) is close to zero, then
  In this case, the ratio depends on the value of the mean noise 〈*n*〉. If the reflection intensities are underestimated, then the mean noise will be negative. When the average noise is close to −〈*I*_t_〉 the ratio will become very large. If the reflection intensities are overestimated, then the mean noise is positive. As 〈*n*〉/〈*I*_t_〉 tends to infinity the ratio will tend towards a value of 1, mimicking twinned data.
(iii) If the mean noise 〈*n*〉 is zero and the variance of the noise var(*n*) is nonzero, then
  In this case, the ratio is always greater than 2 and as var(*n*) increases relative to the true reflection intensity 〈*I*_t_〉 the ratio also increases. At high resolution, where reflection intensities are small relative to the noise, this trend in the ratio towards infinity is often seen.
